# Highly active antiretroviral therapy and hospital readmission: comparison of a matched cohort

**DOI:** 10.1186/1471-2334-6-146

**Published:** 2006-10-05

**Authors:** Bohdan Nosyk, Huiying Sun, Xin Li, Anita Palepu, Aslam H Anis

**Affiliations:** 1 Canadian HIV Trials Network, St. Paul's Hospital, Vancouver, BC, Canada; 2 Centre for Health Evaluation and Outcome Sciences, St. Paul's Hospital, Vancouver, BC, Canada; 3 Department of Healthcare and Epidemiology, University of British Columbia, Vancouver, BC, Canada; 4 Department of Medicine, University of British Columbia, Vancouver, BC, Canada

## Abstract

**Background:**

Despite the known efficacy of highly active antiretroviral therapy (HAART), a large proportion of potentially-eligible HIV-infected patients do not access, and may stand to benefit from this treatment. In order to quantify these benefits in terms of reductions in hospitalizations and hospitalization costs, we sought to determine the impact of HAART on hospital readmission among HIV-infected patients hospitalized at St. Paul's Hospital (SPH) in Vancouver, BC, Canada.

**Methods:**

All patients admitted to a specialized HIV/AIDS ward at SPH (Apr. 1997 – Oct. 2002) were selected and classified as being on HAART or not on HAART based upon their initial admission. Patients were then matched by their propensity scores, which were calculated based on patients' sociodemographics such as age, gender, injection drug use (IDU) status, and AIDS indication, and followed up for one year. Multivariate logistic regression was used to estimate the difference in the odds of hospital readmission between patients on and not on HAART.

**Results:**

Out of a total 1084 patients admitted to the HIV/AIDS ward between 1997 and 2002, 662 were matched according to their propensity score; 331 patients each on and not on HAART. Multivariate logistic regression revealed that patients on HAART had lower odds of AIDS hospital readmission (OR, 0.61; 95% CI, 0.42 – 0.89) compared to patients not on HAART. Odds of readmission among patients on HAART were also significantly lower for non-IDU related readmission (OR, 0.73; 95% CI, 0.53 – 0.99) and overall readmission (OR, 0.72; 95% CI, 0.53 – 0.98).

**Conclusion:**

Propensity score matching allowed us to reliably estimate the association between exposure (on or not on HAART) and outcome (readmitted to hospital). We found that HIV-infected patients who were potentially eligible for, but not on HAART had higher odds of being readmitted to hospital compared to those on HAART. Given the low level of uptake (31%) of HAART observed in our pre-matched hospitalized cohort, a large potential to achieve clinical benefits, reduce hospitalization costs and possibly slow disease progression from improved HAART uptake still exists.

## Background

Despite the known efficacy of highly active antiretroviral therapy (HAART) and its universal coverage in most provinces in Canada, a sizable proportion of HIV-infected patients, particularly subgroups of substance users, do not access HAART [1,2]. Poor uptake, however, is not solely a Canadian problem; evidence from a large study in Europe showed that it is not uncommon to find rates of HAART uptake as low as 59% within observational databases [3], or as low as 30% among injection drug users (IDUs) [4]. A recent review found that HIV-infected individuals who fail to access care or who discontinue HAART have the greatest risk of disease progression and death [5,6].

Uptake and adherence to HAART regimens have also been shown to reduce hospital resource utilization [7-9]. The introduction of HAART in Australia was considered to be the key determinant of a 33% decline in AIDS incidence between 1995 and 1998 [10]. In addition, a study in the United States showed that HAART contributed to a 37% decrease in hospital admissions and a 41% decrease in hospital days [11].

In response to the high levels of resource utilization and costs of care for patients with HIV/AIDS, several studies have focused on identifying predictors of early hospital readmission (within 14 days). This issue was examined among HIV-infected patients with bacterial pneumonia and Pneumocystis Carinii pneumonia. One study prior to the widespread availability of HAART found leaving hospital alone to be a leading risk factor for early readmission, citing this as an important indicator of social isolation [12]. A similar study conducted when HAART was widely available found that female gender, homelessness, residence in a poor neighbourhood, AIDS diagnosis, IDU status, leaving hospital against medical advice (AMA) and previous admission within 6 months was associated with early readmission [13].

Previous studies based on observational data that compared readmission among different cohorts were subject to selection bias. In this study, we use a superior study design by matching our cohorts according to a propensity score to account for potential differences in characteristics between patients who were and were not on HAART. We hypothesized that patients not on HAART would have a higher probability of readmission within one year.

## Methods

### Patient population

All patients admitted to the specialized HIV/AIDS ward at St. Paul's Hospital (SPH), Vancouver, BC, between April 1997 and December 2002 were included in the study. SPH, located in downtown Vancouver, is a major teaching hospital of the University of British Columbia, and has been the main referral center for HIV related diseases since the epidemic began [14]. The specialized HIV/AIDS ward provides comprehensive patient care utilizing infectious disease specialists, nurses, social workers, dietitians, addiction medicine specialists and community physicians. The study received ethical approval from the Providence Health Care Research Ethics Board.

Two databases were used for the study: the SPH Health Records database and the specialized HIV/AIDS ward database. Hospitalization data, provided by the Health Records Department at SPH, included patient demographics, the most responsible diagnosis, IDU status (injection drug use within past 6 months), living status (homeless or not), discharge information (any record of leaving hospital against medical advice (AMA)) and all hospital resource utilization. The AIDS ward database added patient histories of AIDS-defining diagnoses (as listed in table 2), indication of social supports – defined as whether or not the patient had a partner, family or friends – and antiretroviral treatment data.

**Table 1:** Patient Demographics: Pre- and Post- Propensity Score Match

	Pre-propensity score match			Post-propensity score match		
	On HAART	Not on HAART	P-value	On HAART	Not on HAART	P-value
Sample size	342	742		331	331	
Age (Q1 – Q3)	40.8 (36.3, 46.4)	38.4 (33.3, 44.6)	< 0.01	40.8 (36.1, 46.2)	40.8 (35.8, 47.3)	0.68
Female (%)	43 (12.6)	194 (26.2)	< 0.01	43 (13.0)	39 (11.8)	0.64
Social support (%)	255 (74.6)	489 (66.0)	< 0.01	246 (74.3)	242 (73.1)	0.74
Homeless (%)	6 (1.8)	67 (9.0)	< 0.01	6 (1.8)	6 (1.8)	0.52
AIDS (%)	207 (60.5)	458 (61.7)	0.71	205 (61.9)	202 (61.0)	0.81
IDU (%)	85 (24.9)	357 (48.1)	< 0.01	85 (25.7)	82 (24.8)	0.79
AMA (%)	26 (7.6)	132 (17.8)	< 0.01	26 (7.9)	43 (13.0)	0.03

IDU = Intravenous Drug Use, AMA = Indicates leaving hospital against medical advice.

**Table 2:** AIDS-Defining Illnesses at Index Admission: Pre- and Post- Propensity Score Match

	Pre-Propensity Score Match			Post-Propensity Score Match		
	On HAART N (%)	Not on HAART N (%)	P-value	On HAART N (%)	Not on HAART N (%)	P-value

CMV	27 (7.9)	15 (2.0)	< 0.01	27 (8.2)	8 (2.4)	< 0.01
Candidiasis	23 (6.7)	35 (4.7)	0.17	23 (7.0)	17 (5.1)	0.33
Cryptococcis	10 (2.9)	31 (4.2)	0.31	10 (3.0)	14 (4.2)	0.41
HIV Dementia	12 (3.5)	16 (2.2)	0.19	12 (3.6)	15 (4.5)	0.56
Kaposi's Sarcoma	30 (8.8)	24 (3.2)	< 0.01	30 (9.1)	17 (5.1)	0.05
Lymphoma	17 (5.0)	20 (2.7)	0.06	17 (5.1)	14 (4.2)	0.58
MAC	29 (8.5)	28 (3.8)	< 0.01	29 (8.8)	12 (3.6)	0.87
PCP	84 (24.6)	135 (18.2)	0.02	83 (25.1)	68 (20.1)	0.85
Pneumonia	29 (8.5)	83 (11.2)	0.17	29 (8.8)	30 (9.1)	0.89
Toxoplasmosis	6 (1.8)	8 (1.1)	0.36	6 (1.8)	3 (0.9)	0.78
Tuberculosis	8 (2.3)	27 (3.6)	0.26	8 (2.4)	10 (3.0)	0.79
Wasting	24 (7.0)	36 (4.9)	0.15	24 (7.3)	19 (5.7)	0.66

CMV = Cytomegalovirus, MAC = Mycobacterium Avium Complex, PCP = Pneumocystis Carinii Pneumonia.

### Study design

Each patient's first admission to the HIV/AIDS ward during the time period was defined as the index admission, at which time patients were categorized as being "on HAART "or "not on HAART". Patients who had ever had an AIDS/Advanced HIV diagnosis (ICD-9: 42.0–44.9) reported up to their index admission were classified as having AIDS. Patients who died at index admission were omitted from the analysis.

Patients were classified as being readmitted if they were admitted anywhere within the hospital during one year of follow-up. To explore the patterns of readmission, the most responsible diagnosis of readmission was reviewed. Overall readmission was compared between cohorts, however the cause some hospital admissions may have been for reasons other than patients' HIV/AIDS status. Thus, readmitted patients were further classified in two additional analyses: 1) being readmitted for AIDS diagnoses (defined as having been admitted to the AIDS ward with an AIDS/advanced HIV diagnosis); and 2) being readmitted for non-IDU reasons if they were ever admitted to the hospital with a most responsible diagnosis not related to IDU illness during the one-year follow-up. We chose to examine non-IDU related readmission in order to separate out the effects of intravenous drug use on hospital resource utilization, which is assumed to be independent of any effect that HAART might have. Non-IDU related admissions include those for AIDS, as well as other admissions not a result of intravenous drug use.

### Statistical analysis

Univariate comparison between the two cohorts (on HAART vs. not on HAART) was performed using the Wilcoxon rank-sum tests for continuous variables and contingency tables for categorical variables.

A propensity score was used to balance baseline characteristics between the two groups [15,16]. More specifically, we fitted a logistic regression model to calculate the propensity score, based on patients' sociodemographics, such as age, gender, injection drug use (IDU) status, and AIDS indication. Each patient on HAART was then matched to the patient not on HAART with the closest propensity score (up to 5-digit matching).

Multivariate logistic regression models, adjusting for the propensity score and AMA at index admission, were used to explore the effects of HAART within the three separate analyses listed above. The AMA variable was expected to be associated with hospital readmission, but not with being on HAART, and was therefore left out of the propensity score match. All statistical analysis was performed using the SAS 8.2 software program (SAS Institute, Inc., Cary, NC).

## Results

A total of 1150 patients – 358 (31%) on HAART and 792 (69%) not on HAART – were admitted to the HIV/AIDS ward between 1997 and 2002. After excluding deaths at index admission, we had a total of 1084 patients – 342 on HAART and 742 not on HAART for the analysis. Patient sociodemographics at index admission are presented in Table 1. Gender, age, and IDU status were significantly different between those on and not on HAART. In addition, homelessness was far more prevalent among subjects not on HAART, as were instances of leaving hospital against medical advice. Social supports were significantly greater among those on HAART. The demographics of the 662 matched patients at index admission are shown in the second set of columns in Table 1. As expected, matched variables including the proportion of patients with indication of AIDS, IDU status and homelessness were not statistically significantly different between those on and not on HAART.

Table 2 lists all of the previously-indicated AIDS-defining illnesses of patients in each cohort at index admission pre- and post-propensity score match. It was shown that before matching, the proportions of patients who had ever have CMV, MAC, Lymphoma, Kaposi's sarcoma, and PCP differed significantly between the two groups. However, the differences on majority of the illnesses were not statistically significantly different following the propensity score matching procedure.

Table 3 lists the results from univariate analysis. The number of patients readmitted for AIDS diagnoses was not significantly different between the two groups (76 vs. 95, p = 0.09). Among those without previous indication of AIDS, patients on HAART were significantly less likely to be readmitted for AIDS diagnoses than those not on HAART (3 (2.4%) vs. 18 (14%), p = < 0.01). Also, significantly fewer patients on HAART were readmitted for non-IDU related reasons (141 vs. 168, p = 0.04) and overall (149 vs. 181, p = 0.01).

**Table 3:** Readmissions in the One-Year Follow-Up Period

	On HAART N (%)	Not on HAART N (%)	P-value
Sample size	331	331	
Readmitted for AIDS diagnosis	76 (23.0)	95 (28.7)	0.09
*With previous AIDS indication*^*1*^	73 (35.6)	77 (38.1)	0.60
*Without previous AIDS indication*^*2*^	3 (2.4)	18 (14.0)	< 0.01
Readmitted for non-IDU related reasons	141 (42.6)	168 (50.8)	0.04
Overall readmitted	149 (45.0)	181 (54.7)	0.01

1. On HAART: n = 205, not on HAART: n = 202.

2. On HAART: n = 126, not on HAART: n = 129.

Table 4 presents the results from the multivariate logistic regression models. We found that after adjusting for the propensity score and AMA indication at index admission, patients on ART faced lower odds of being readmitted for AIDS diagnosis (OR 0.66; 95% CI: 0.46 – 0.96), as well as non-IDU related readmission (OR 0.71; 95% CI: 0.52 – 0.97). Finally, odds of overall readmission were lower among patients on HAART (OR 0.70; 95% CI: 0.51 – 0.95). Leaving hospital against medical advice (AMA variable) was a significant predictor of readmission overall (OR: 2.29, 95% CI: 1.31–4.01) for non-IDU related reasons (OR: 2.15, 95% CI: 1.22–3.79), but not for AIDS diagnoses (OR: 1.38, 95% CI: 0.69–2.78).

**Table 4:** Results from Multivariate Logistic Regression Models

Multivariate Logistic Regression	On HAART vs. Not on HAART	AMA vs. Not AMA
	Odds Ratio (95%C.I.)	Odds Ratio (95%C.I.)
*AIDS readmission*^*1*^		
Not Readmitted	1	1
Readmitted for AIDS-related illness	0.66 (0.46, 0.96)	1.38 (0.69, 2.78)
Readmitted for other reasons	0.74 (0.50, 1.10)	3.39 (1.84, 6.24)
		
*Non-IDU related readmission*^*1*^		
Not Readmitted	1	1
Readmitted for non-IDU related reasons	0.71 (0.52, 0.97)	2.15 (1.22, 3.79)
Readmitted for IDU-related reasons	0.54 (0.21, 1.37)	4.02 (1.37, 11.86)
		
*Overall readmission*		
Not Readmitted	1	1
Readmitted	0.70 (0.51, 0.95)	2.29 (1.31, 4.01)

1. Multinomial, multivariate logistic regression.

## Discussion

In this study, we applied propensity score methodology to investigate the impact of HAART on hospital readmission. Our results showed that HAART reduces the odds of hospital readmission, primarily for diagnoses of HIV-related illness, including AIDS-defining diagnoses. The advantages of using propensity score matching in observational epidemiological studies have been addressed elsewhere [20]. Generally speaking, treated and non-treated cohorts in observational studies may differ on a number of baseline covariates; ignoring these differences may confound the treatment effect, thus biasing estimates of treatment effect. The propensity score method allowed us reduce selection bias by minimizing differences in patients' observed covariates in the comparison of those on and not on treatment and therefore provided more reliable estimates of treatment effect.

Controlling for AIDS indication, gender and IDU status, which are supported within the literature as univariate predictors of being treated with HAART [18-20], patients on HAART had significantly lower odds of being readmitted to hospital for AIDS diagnoses. This result was found regardless of the fact that patients on HAART had (marginally) more AIDS diagnoses at index admission, suggesting that this cohort was slightly sicker, and would require more hospitalizations. Further analysis using Cox regression showed that, controlling for leaving hospital AMA, time to readmission was found to be statistically significantly shorter among those not on HAART (results not presented). Our results are conservative, given the possibility that some patients may have taken up, or dropped out of treatment in the 'not on HAART' and 'on HAART' cohorts, respectively. Furthermore, we found that patients who had not previously been diagnosed with AIDS and were not on HAART were significantly more likely to be readmitted for an AIDS diagnosis than those not previously diagnosed with AIDS who were on HAART. This result suggests more rapid disease progression among patients not on HAART during our one-year follow-up period; a result also supported by existing literature [6].

We found that a relatively high proportion of patients in each group were readmitted to hospital after index admission (on HAART = 42%, not on HAART = 51%). This is high in comparison to Sherer et al., who found that only 25% of a patient population that had visited an urban public hospital HIV clinic was hospitalized in a given year [21]. The possible explanation for the difference is that our study population may be at a more advanced disease stage, as our cohort included high proportion of patients with AIDS and the cohort was followed after an inpatient visit, rather than outpatient visit which was indicated in Sherer et al. study. In addition, under Canadian healthcare system, inpatient care was free of charge, which may differ from studies conducted in different healthcare settings. Publicly funded health system might induce patients to use more health care resources, including visiting hospital more often. As a result, the probability of being admitted might be higher than those under other healthcare settings.

Our analysis also highlighted the fact that an alarming proportion of HIV-infected patients admitted to hospital were not being treated with HAART. Only 31% of patients admitted to the specialized AIDS ward from 1997–2002 were on HAART. Low rates of uptake were also observed in a cohort of ICU-presenting HIV/AIDS patients in Italy [22]. This recent study found that hospitalization patterns before and after the introduction of HAART had not changed, primarily due to a flat rate of ART uptake between 1994 and 1998. Physician discretion [23], discontinuation due to drug toxicity or intolerance [24,25] and patient attitudes against the efficacy of antiretroviral therapy [16,26-28] have all been indicated as reasons why patients may not choose HAART.

The following limitations merit discussion. First, CD4 count at index admission was not controlled for in the baseline analysis. The causal relationship between CD4 and HAART initiation is unclear and time of HAART initiation in our population is unknown. In addition, the objective of HAART therapy is to increase CD4 count, and thus reduce the onset of AIDS-defining illnesses and other disease which require inpatient care. CD4 count is therefore an intermediate variable in the causal pathway between the exposure (HAART) and outcome (hospital readmission); inclusion of this covariate would constitute one type of overmatching, which is to be avoided [29]. Second, although we only observed inpatient care at a single institution, given its central geographical location and the comprehensive and specialized care available for this population at SPH, it is unlikely that our patient population sought care elsewhere in the follow-up period. Finally, treatment history, disease duration and the reasons patients were not on HAART were unknown. We believe that the use of the propensity score method to control for the existence of imbalanced covariates between our case and control cohorts provided us with the most unbiased estimates of treatment effect possible and limited the impact of the omission of these variables.

## Conclusion

HIV-infected patients who were not on HAART were readmitted to hospital more frequently and therefore utilized more hospital resources overall compared to those on HAART. The implications of these results are important to both physicians and health care administrators. From the physician's perspective, low uptake and the evidence of its consequences on disease progression in a matched cohort of patients highlights the need for healthcare providers to engage eligible HIV-infected persons in a frank discussion addressing the barriers to HAART that many patients face. From an administrative perspective, although antiretroviral drug costs may be considerable, our study provides some evidence that these costs will be offset by decreased hospitalization costs.

## Competing interests

The author(s) declare that they have no competing interests.

## Authors' contributions

BN took part in the conception of the study design and interpretation of results and drafted the manuscript, HS undertook statistical analyses and took part in conceiving the study design and interpreting results, XL took part in conceiving the study design and interpreting results, AP was involved in the critical revision of the article for important intellectual content, AA was involved in the critical revision of the article for important intellectual content. All authors read and approved the final manuscript.

## Pre-publication history

The pre-publication history for this paper can be accessed here:


